# Multicenter, retrospective cohort study of antimycobacterial treatment-related harms among patients with non-tuberculosis *Mycobacterium* infections in the United States

**DOI:** 10.1128/aac.01596-24

**Published:** 2025-03-04

**Authors:** Michael P. Veve, Rachel M. Kenney, Alisar M. Aljundi, Michelle S. Dierker, Vasilios Athans, Anita B. Shallal, Nimish Patel

**Affiliations:** 1 Department of Pharmacy, Henry Ford Hospital 24016 , Detroit, Michigan, USA; 2 Department of Pharmacy Practice, Eugene Applebaum College of Pharmacy and Health Sciences, Wayne State University 15538 , Detroit, Michigan, USA; 3 Department of Pharmacy, Hospital of the University of Pennsylvania 21798 , Philadelphia, Pennsylvania, USA; 4 Department of Infectious Diseases, Henry Ford Hospital 24016 , Detroit, Michigan, USA; 5 Skaggs School of Pharmacy and Pharmaceutical Sciences, University of California, San Diego 15500 , La Jolla, California, USA; University of Pittsburgh School of Medicine, Pittsburgh, Pennsylvania, USA

**Keywords:** non-tuberculosis mycobacteria, *Mycobacterium abscessus*, adverse drug event, antimicrobial stewardship

## Abstract

Non-tuberculosis mycobacteria (NTM) are extensively drug-resistant organisms that require long-term therapy. The study purpose was to quantify the incidence of and risk factors for antimycobacterial-associated adverse drug events (ADEs) in persons with NTM infections receiving outpatient therapy. A multicenter, retrospective cohort was performed of persons with NTM infections who received antimycobacterial treatment from 2013 to 2024. Inclusion criteria were age ≥18 years, ≥1 month of outpatient treatment, and ≥1 follow-up outpatient visit within 3 months of index encounter. *Mycobacterium avium* complex and *Mycobacterium tuberculosis* complex were excluded. The primary outcome was development of pre-specified treatment-related ADE or acute kidney injury (AKI), thrombocytopenia, and/or *Clostridioides difficile* infection (CDI) through 12 months of therapy. Secondary outcomes included therapy discontinuation due to any treatment-related ADEs. Two hundred patients were included: 14% developed a pre-specified ADE. *Mycobacterium abscessus* (29%) was the most common pathogen; most initial regimens included a macrolide (54%), systemic aminoglycoside (24%), β-lactam (24%), or tetracycline derivative (22%). The most common pre-specified ADEs were thrombocytopenia (9%), AKI (8%), and CDI (<1%). The median (IQR) time-to-ADE was 25 (18–38) days from initial outpatient regimen; patients who received aminoglycoside- or oxazolidinone-based therapies were more likely to develop a pre-specified ADE (adjOR, 3.9; 95% CI, 1.7–9.2). Therapy discontinuation due to any ADE occurred in 35% of patients; the median (IQR) time-to-any ADE was 32 (21–58) days. ADEs in persons with NTM infections are common and occur near the first month of outpatient treatment. Intensified monitoring and/or use of more tolerable antimycobacterial regimens early in treatment may be an appropriate approach to avoid harms.

Treatment of non-tuberculosis mycobacteria is complicated by adverse drug events (ADEs). This work quantified the incidence and time course of pre-determined, clinically relevant ADEs (acute kidney injury, thrombocytopenia, and *C. difficile* infection), which occurred in 14% of patients within 30 days of outpatient treatment.

## INTRODUCTION

Non-tuberculosis mycobacteria (NTM) are naturally occurring organisms found in water and soil that have the potential to cause extensively drug-resistant and difficult-to-treat infections in humans (1–4). There were an estimated 181,000 annual pulmonary NTM infections in the United States during 2014; non-pulmonary NTM infections are relatively understudied, though the prevalence of all NTM infections is expected to be increasing as the population ages (2). While the absolute number appears low, NTM infections are associated with considerable healthcare expenditures, resulting in 1.7 billion dollars of related healthcare costs (2). The majority of these costs are attributed to the intensive healthcare needs associated with effectively managing NTM infections (4, 5).

Due to the chronic and persisting nature of NTM infections, long-term antimycobacterial treatment is often necessary to achieve cure (1). However, the limited availability of drugs with activity against NTM and the propensity for several species to harbor complicated resistance mechanisms present a major challenge to effective treatment (1). Treatment-related toxicities and intolerabilities of conventional antimycobacterials are common and further complicate treatment (4, 6). Intravenous (IV) aminoglycosides are frequently used in initial NTM anti-mycobacterial regimens but are associated with kidney injury and ototoxicity when used long-term, even at low doses (7, 8). β-Lactams with NTM activity, such as imipenem/cilastatin, ceftazidime, or cefoxitin, are dosed several times per day and are only available IV (8). Additionally, IV antimycobacterials require long-term vascular access, which increases the risk for catheter-related complications, such as occlusion, dislodgement, infection, or thrombosis (9). Several oral antimycobacterial drug classes, such as sulfonamides, macrolides, oxazolidinones, and fluoroquinolones, are associated with notable adverse effects or drug–drug interactions that are more frequent with long-term use (8). Many antimycobacterials are broadly capable of disrupting bowel flora and are subsequently associated with *Clostridioides difficile* infection (CDI), which may contribute to patient morbidity. Additionally, NTM infections disproportionately impact elderly and immunocompromised patients with comorbid conditions, which adds to treatment complexity and the likelihood of antimycobacterial complications (5). Virtually all conventional antimycobacterials used to treat NTM infections are associated with the potential for serious adverse events, which can contribute to patient morbidity and mortality (6, 8).

Limited data are available to adequately describe and compare NTM treatment regimens or the potential role of novel agents with more desirable drug properties. The aim of this study was to quantify and characterize pre-specified, objective adverse events associated with NTM therapy regimens. Specifically, the study objectives were to (i) quantify the incidence of and (ii) determine the risk for adverse events associated with NTM therapy. A better understanding of the relevance and frequency of antimycobacterial harms can help clinicians determine when conventional therapies should be deferred in favor of agents with a lower likelihood of causing deleterious effects.

## MATERIALS AND METHODS

### Study design

This was an observational, multicenter, retrospective cohort study of persons with NTM infections in the United States who received antimycobacterial treatment for at least 1 month and were assessed for up to 12 months from index NTM visit. Patients were derived from three geographically distinct U.S. metropolitan areas with a high prevalence of NTM infections (2): Henry Ford Health (Detroit, MI), University of California at San Diego Health (San Diego, CA), and the Hospital of the University of Pennsylvania (Philadelphia, PA). These sites are contained within a larger consortium of health systems with extensive ambulatory clinic networks that treat NTM patients.

The inclusion criteria were as follows: (i) age ≥18 years; (ii) diagnosis code consistent with NTM infection according to the U.S. International Classification of Diseases, 10th revision, diagnosis coding system (i.e., A31.xx); (iii) present history of microbiologically confirmed NTM infection, including pulmonary disease (1); (iv) receipt of antimycobacterial treatment for NTM infection for at least 1 month in the outpatient setting; and (v) receipt of treatment from January 2013 to June 2024. Hospitalized patients who received antimycobacterial therapy as an inpatient during index diagnosis but were transitioned to outpatient care were included. Patients with human immunodeficiency virus diagnosis, infection only with *M. avium* complex (MAC) or *M. tuberculosis* complex, or patients without at least one follow-up outpatient visit or home health assessment within the 3 months following index NTM visit were excluded. *M. tuberculosis* complex and MAC were excluded due to more well-defined antimycobacterial regimens and harms data that may not be reflective of treatment challenges related to other NTM species. Patients who met criteria for inclusion were only included once.

### Outcomes and key study definitions

The primary study outcome was development of pre-specified, objective antimycobacterial-related ADEs. The components of the pre-specified ADEs were objectively defined to avoid information bias that could lead to misclassification of event status. The composite of pre-specified, objective ADE included acute kidney injury (AKI), CDI, and/or thrombocytopenia and were determined using laboratory values and/or testing. Acute kidney injury was defined using the Kidney Disease Improving Global Outcomes (KDIGO) criteria (10). Serial creatinine values were collected to determine changes in values associated with AKI; patients receiving hemodialysis at baseline were not eligible for the AKI ADE case definition. CDI was defined using the IDSA/SHEA guidelines; the case definition was limited to individuals with *C. difficile* identified from a stool sample (whichever method of detection used at the local site) accompanied by ≥3 unformed stools in a 24 h period with other causes of diarrhea ruled out (11). Thrombocytopenia was captured based on serial platelet readings and compared with pre-treatment platelet values. Thrombocytopenia was defined as a platelet decline of ≥50% from baseline laboratory value (12). Baseline laboratory values related to the pre-specified, objective ADEs were recorded prior to or at the start of the outpatient antimycobacterial therapy, depending on which values were available.

The utilization of pre-specified, objective ADE definitions may limit the total number and types of other potential ADEs patients experience while receiving antimycobacterial treatment. As such, a secondary ADE assessment was performed and included the development of any antimycobacterial ADE that resulted in treatment discontinuation. Treatment discontinuation was defined as any discontinuation of an individual or multiple antimycobacterials (i.e., any change in regimen drug selection) due to an adverse event, as documented from NTM treatment healthcare provider notes placed in the electronic health record (EHR) throughout the duration of therapy. Only ADEs that resulted in treatment discontinuation were evaluated in order to quantify more relevant events or toxicities in the absence of more formal, objective ADE definitions. Additionally, ADE treatment discontinuation did not include modifications to antimycobacterial dosing due to the variety of non-ADE factors that can prompt a decision to adjust antimycobacterial dosing, such as therapeutic drug monitoring or changes in organ function. An additional secondary outcome included the proportion of patients who experienced any ADE (i.e., objective and subjective) that resulted in an ED or hospitalization event, as documented in the EHR. Patients who experienced IV antibiotic access complications were included in the secondary outcome definition of any ADE that resulted in an ED visit or hospitalization.

The “time zero” for assessing ADEs, including pre-specified and any reported ADE that resulted in treatment discontinuation, was from the date of outpatient antimycobacterial therapy initiation; in the scenario where the index infection was hospital admission-associated, the treatment-related ADE definition was only assessed after the patient was discharged to reflect a more pragmatic approach for monitoring antimycobacterial drug therapy (i.e., lack access to frequent laboratory values). ADE outcomes were assessed for up to the prescribed duration of antimycobacterial therapy for a maximum of 12 months after “time zero,” depending on follow-up clinic laboratory value availability.

Patient and infection characteristics were collected from the EHR and included demographics, comorbidities, laboratory findings, NTM classification (1) and microbiology, antimycobacterial therapy selection, including subsequent NTM treatment regimens after an ADE, and patient outcomes. Clinical and demographic characteristics included sex, race, age, insurance status, and select comorbid conditions. Autoimmune disease was defined as rheumatoid arthritis, systemic lupus erythematosus, inflammatory bowel disease, multiple sclerosis, diabetes mellitus type 1, Guillain–Barré syndrome, chronic inflammatory demyelinating polyneuropathy, and psoriasis. Antimycobacterial dosing strategies for therapies known to have a significant adverse effect profile were also collected. NTM treatment outcomes included culture clearance in respiratory infections and death while on therapy.

### Data source

Screening data were extracted from each site’s EHR using the Epic SlicerDicer tool (Epic Systems Corporation, Verona, WI, USA). Each site investigator was trained on data extraction methods using a pre-recorded video to create a consistent screening process. Data extraction parameters were set to identify patients with NTM infections during the study timeframe using SlicerDicer International Classification of Diseases, 10th revision, grouper terms (i.e., A31.xx), while excluding patients with a history of *M. tuberculosis* and *M. avium* complex. After screening patients for inclusion, data variables were extracted from the EHR by manual chart review, transcribed to an electronic case report form (REDCap, Vanderbilt University, Nashville, TN, USA), and hosted on secure internal servers.

### Statistical analysis

Descriptive statistics were used to characterize the population and frequency of toxicities. Categorical variables were compared with Pearson’s χ or Fisher’s exact test; continuous variables were compared with the Mann–Whitney U-test. Stratified analyses were performed to assess the presence/absence of effect modification. To determine the patients who have the greatest likelihood of experiencing a pre-specified, objective ADE (i.e., AKI, thrombocytopenia, CDI), variables associated with this outcome (*P* < 0.2) in bivariate analyses or with clinical rationale were entered into a multivariable logistic regression model using a backwards-stepwise approach. Categorical variables were evaluated for collinearity using the χ test prior to model inclusion. Model fit was assessed using the Hosmer–Lemeshow test. All statistical analyses were performed using SPSS Software for MacIntosh v.29.0 (IBM Corp, Armonk, NY).

## RESULTS

A total of 200 patients were included; 28 (14%) developed a pre-specified, objective ADE. The most common pre-specified ADEs were thrombocytopenia (17, 9%), AKI (15, 8%), and CDI (1, <1%); baseline characteristics of patients who did and did not develop a pre-specified, objective ADE are listed in Table 1. Among patients who developed AKI, the most common KDIGO class was 1 (13, 87%) and 3 (2, 13%). In the overall patient cohort, the majority (151, 76%) of patients had NTM treatment initiated in an outpatient clinic setting; 49 (25%) patients had initial NTM diagnosis during a hospitalization and were transitioned to outpatient therapy. One hundred ninety (95%) patients had health insurance at the index infection diagnosis. The most common specialty consultants involved in NTM management included infectious diseases (188, 94%), pulmonology (77, 39%), dermatology (42, 21%), and various surgery specialties (36, 18%). Seventy-three (37) patients received surgical intervention in addition to antimycobacterial treatment for disease management.

**TABLE 1:** Baseline and infection characteristics of patients treated for non-tuberculosis mycobacterial infections who developed a pre-specified adverse drug event^*b*^

Characteristic *n* (%) or median (IQR)	Antimycobacterial ADE^*a*^ *n* = 28	No ADE *n* = 172	*P*-value
Patient characteristics			
Age, years	62 (58–73)	61 (49–70)	0.20
Male sex	17 (61%)	96 (56%)	0.69
White/Caucasian race	22 (79%)	105 (61%)	0.091
Previous NTM treatment experienced	0	7 (30%)	0.20
Prior hospitalization, 6 months	11 (39%)	32 (19%)	0.023
Prior surgical procedure, 1 month	9 (32%)	27 (16%)	0.059
Previous antimicrobial exposure, 3 months	19 (68%)	76 (44%)	0.025
Comorbid conditions			
Tobacco use	3 (11%)	12 (7%)	0.49
Autoimmune disease	10 (36%)	25 (15%)	0.006
Gastroesophageal reflux	10 (36%)	65 (38%)	1.0
Chronic obstructive pulmonary disease	8 (29%)	38 (22%)	0.45
Interstitial lung disease	1 (4%)	13 (8%)	0.70
Ventricular assist device	0	7 (4%)	0.60
Moderate/severe renal disease	8 (27%)	29 (17%)	0.14
Cystic fibrosis	0	5 (3%)	1.0
Splenectomy	0	2 (1%)	1.0
Solid organ transplant recipient	3 (11%)	14 (8%)	0.71
Cytotoxic chemotherapy, 3 months	2 (7%)	8 (5%)	0.63
Chronic steroid use, 3 months	2 (7%)	11 (6%)	1.0
Infection characteristics			
NTM disease type, non-inclusive			
Nodular	8 (29%)	37 (22%)	0.41
Fibro-cavitary	3 (11%)	5 (3%)	0.09
Other pulmonary	4 (14%)	35 (20%)	0.45
Extra-pulmonary	0	2 (3%)	1.0
Non-pulmonary	15 (54%)	105 (58%)	0.65
Non-pulmonary NTM disease			
Skin and skin structure	8 (53%)	56 (53%)	0.85
Bone and joint	3 (20%)	11 (11%)	0.39
Intra-abdominal	4 (27%)	17 (17%)	0.47
Other	0	15 (15%)	0.21

^*a*^ Includes acute kidney injury, thrombocytopenia, or *C. difficile* infection only.

^*b*^ NTM, non-tuberculosis mycobacteria.

There were 217 organisms isolated: the most commonly isolated organism was *M. abscessus* (64, 29%), followed by *M. chelonae* (39, 18%), *M. fortuitum* (25, 12%), *M. kansasii* (21, 10%), *M. marinum* (14, 6%), and other species (54, 25%). Of the 85 (42%) patients with a pulmonary NTM infection, only 22 (26%) had documented culture conversion. Mycobacteria were most commonly isolated from respiratory (69, 35%), wound (32, 16%), tissue (23, 11%), and fluid (18, 9%) specimens.

Most initial NTM regimens included a combination of a macrolide (107, 54%), systemic aminoglycoside (47, 24%), β-lactam (47, 24%), tetracycline derivative (44, 22%), or oxazolidinone (40, 20%). The other antimycobacterial agents initially prescribed were most commonly a rifamycin (34, 17%), ethambutol (38, 19%), fluoroquinolones (38, 19%), trimethoprim/sulfamethoxazole (29, 15%), isoniazid (10, 5%), and clofazimine (4, 2%). There were four patients who received inhaled amikacin.

Regarding select antimycobacterial dosing strategies, aminoglycosides were most commonly weight-based and dosed daily, followed by thrice weekly; linezolid was most commonly dosed at 600 mg twice daily, followed by 600 mg daily; eravacycline was most commonly dosed twice daily. One hundred forty (70%) patients received initial therapy with all oral agents; however, 3/140 (2%) patients also received concomitant inhaled amikacin. Patients infected with *M. abscessus* less commonly received initial therapy with all oral agents (22 [34%] vs 118 [87%], *P* < 0.001). The median (IQR) total duration of observed NTM therapy was 181 (69–356) days; 45 (22%) patients received therapy for longer than 12 months. Patients received a median (IQR) of 2 (1–3) unique NTM regimens, and 13 (7%) patients died while receiving NTM treatment. Antibiotics with NTM activity that have been recently Food and Drug Administration approved were not commonly utilized early in treatment regimens: bedaquiline (1, 0.5%), inhaled liposomal amikacin (3, 1%), eravacycline (4, 2%), omadacycline (6, 3%), tedizolid (5, 3%), and tigecycline (11, 5%).

Of the 28 patients who developed a pre-specified, objective ADE (i.e., thrombocytopenia, AKI, or CDI), the median (IQR) time to event was 25 (18–38) days from initial outpatient treatment regimen. There were no patients who developed multiple ADEs when using the primary ADE definition, but five patients developed an additional ADE that resulted in treatment discontinuation while receiving another NTM regimen. Twenty-four patients who developed a pre-specified ADE had treatment discontinued and were initiated on a new NTM regimen. Patients with *M. abscessus* infection more frequently developed a pre-specified, objective ADE compared with those without *M. abscessus* infection (14 [22%] vs 14 [10%], *P* = 0.047). Additionally, patients who received initial NTM therapy with a systemic aminoglycoside (16 [34%] vs 12 [8%], *P* < 0.001) more commonly developed a pre-specified, objective ADE. Patients who received initial therapy with an oxazolidinone (9 [23%] vs 19 [12%], *P* = 0.12) or β-lactam (11 [23%] vs 17 [11%], *P* = 0.052) did experience numerically higher ADE, but these results were not statistically significant. Conversely, patients who received an initial regimen with all oral agents less frequently developed a pre-specified, objective ADE when compared with patients who initially received an IV-containing regimen (10 [7%] vs 18 [30%], *P* < 0.001); patients who received an initial regimen with all oral agents were also less frequently infected with *M. abscessus* when compared with patients who initially received an IV-containing regimen (22 [16%] vs 42 [70%], *P* < 0.001). Patients who did not receive an initial regimen with oral agents but developed an ADE more commonly received a subsequent regimen with all oral agents (*P* < 0.001).

Results of the bivariate analyses and clinical rationale dictated the variables selected for inclusion into the multivariable regression model for the primary ADE outcome of AKI, CDI, or thrombocytopenia: receipt of initial regimen with an aminoglycoside or oxazolidinone and autoimmune disease (Table 2). Other variables were excluded from the model because of unmet clinical or statistical criteria to preserve the *n*:k ratio or to prevent inclusion of variables that may be colinear. In the final parsimonious model, receipt of the initial regimen with an aminoglycoside or oxazolidinone and autoimmune disease was independently associated with an increased odds of developing a pre-specified, objective ADE.

**TABLE 2:** Variables associated with antimycobacterial adverse drug events, including a composite of acute kidney injury, thrombocytopenia, or *C. difficile* infection

Variable	unAdjOR (95% CI)	*P*-value	AdjOR (95% CI)
Receipt of initial regimen including an aminoglycoside or oxazolidinone	4.0 (1.7–9.3)	<0.001	3.9 (1.7–9.2)
Autoimmune disease	3.3 (1.4–7.9)	0.006	3.1 (1.2–7.8)
Receipt of prior antimicrobial therapy within 3 months^*a*^	2.7 (1.1–6.2)	0.02	Not tested
*M. abscessus* infection^*a*^	2.4 (1.1–5.5)	0.028	Not tested

^*a*^ Not included in final multivariable model due to collinearity.

^*b*^ Hosmer–Lemeshow goodness-of-fit test results: *P* = 0.461.

There were 70 (35%) patients who developed an ADE that led to discontinuation of NTM therapy; the majority of patients were initiated on a new NTM treatment regimen. Excluding AKI, thrombocytopenia, or CDI as previously presented, the types of ADEs that led to drug discontinuation were gastrointestinal intolerance (25, 36%), rash (8, 11%), ototoxicity (6, 9%), drug interaction resulting in critical laboratory values (2, 3%), hyperkalemia (2, 3%), dizziness (2, 3%), ocular toxicity (2, 3%), deep vein thrombosis due to peripherally inserted central catheter placement for IV NTM therapies (1, 1%), inability to self-administer home IV antibiotics (1, 1%), and dysglycemia (1, 1%). The median (IQR) time to developing an ADE that led to discontinuation of NTM therapy was 32 (21–58) days. Eight (11%) patients required an ED visit or hospitalization for ADE management, which were most commonly laboratory abnormality-related or an adverse effect from IV antibiotic access. In bivariate analysis, patients who developed an ADE that resulted in treatment discontinuation were more likely to be >56 years old (unAdjOR, 2.3; 95% CI, 1.2–4.3), infected with *M. abscessus* (unAdjOR, 2.1; 95% CI, 1.1–3.9), receive an initial NTM regimen containing a systemic aminoglycoside or oxazolidinone (unAdjOR, 4.3; 95% CI, 2.3–7.9), and receive an initial NTM regimen containing a β-lactam (unAdjOR, 3.5; 95% CI, 1.8–6.7).

## DISCUSSION

This study found that among a cohort of persons infected with non-MAC NTM, the incidence of antimycobacterial-related AKI, CDI, or thrombocytopenia was 14% and generally occurred within the first month of outpatient treatment. Patients who received initial therapy with an aminoglycoside or oxazolidinone antibiotic were significantly more likely to develop an ADE, although these patients were also more commonly infected with *M. abscessus* that is usually resistant to other oral options that can necessitate the use of alternative, more toxic therapies (1, 4, 8). Patients who received all oral antimycobacterial regimens less frequently developed a pre-specified ADE, at more than half the frequency compared to IV-containing regimens, but were also more commonly infected with *Mycobacterium* spp. that can be treated with agents possessing a safer drug profile. Additionally, one in three patients experienced any ADE that resulted in drug discontinuation and most commonly included gastrointestinal intolerance.

While there is guidance detailing adverse reaction risk potential related to the therapies used to treat NTM infections (13–15), there are extremely limited data describing the true incidence of toxicities in NTM-treated patients. Grimes and colleagues evaluated 29 solid organ transplant recipients infected primarily with MAC organisms (43.3% MAC, 24% *M*. *abscessus*) (16). Over 90% of patients developed an adverse event while on therapy (i.e., drug toxicity, clinically significant drug interaction), and 57% required antimycobacterial discontinuation, compared with 36% in the present study. Gastrointestinal intolerance was the most frequently reported ADE (41%), followed by ototoxicity (36%) and visual disturbances (29%); no patients developed AKI, thrombocytopenia, or CDI. Grimes and colleagues observed a higher overall prevalence of ADEs and ADEs requiring treatment discontinuation compared with the present study, which could represent differences in ADE endpoint definitions, patient populations, monitoring, or small sample size. Tsai and colleagues performed a retrospective cohort study of NTM-infected patients and evaluated factors associated with favorable treatment outcome (17). A total of 98 patients received treatment, where 67% of cases were attributed to MAC and 18% to *M. abscessus*. About 37% of patients who received antimycobacterial treatment developed an ADE, which was most commonly gastrointestinal intolerance (39%). Within this cohort, 3% of patients were reported to have AKI; there were no reports of thrombocytopenia or CDI. Overall, the ADE data from these two studies have similarities to the present study, but variability in NTM population, treatment selection, and intensity of monitoring could have resulted in outcome differences (16, 17).

While this study provides needed context into the management of NTM patients, there are several notable limitations. A retrospective design was selected given the scarcity of NTM infections in the United States and their associated ADEs. Due to the observational nature of this study, the ADE risk factor analysis approach is limited in ability to assess event causality; however, all events were temporally related to antimycobacterial therapy. A standardized electronic case report form and data dictionary were utilized across the three study sites to decrease misclassification bias. Additionally, the primary ADE outcome of AKI, CDI, or thrombocytopenia was evaluated using laboratory values and not subjective reports; as such, these data are likely an underestimate of the true ADE harm potential of NTM treatment. These data are also likely to be under-reported as patients may have a higher likelihood of seeking care at a local hospital where abnormal laboratory assessment would have been recorded and potentially missed during retrospective review. The multicenter nature of this study was necessary to evaluate a sufficient patient size, but differences in health system management and intensity of monitoring for persons with NTM infections may have contributed to inherent differences in outcome. Specific dosing recommendations for aminoglycoside- and oxazolidinone-based therapies may differ by institution, where more conservative dosing has been previously associated with fewer toxicity in NTM (8). Furthermore, the study timeframe reflects a period in which several new therapies with NTM activity were newly developed and may not accurately capture their contemporary use. The patient cohorts included heterogeneous NTM organisms and infection types (i.e., predominantly non-pulmonary) that may limit the application of study findings, but these patients received treatment regardless, and these data have important implications on treatment parameters and drug selection. Patients infected with MAC or *M. tuberculosis* were purposely excluded as they represent populations with both robust treatment selection recommendations and ADE outcomes data.

### Conclusion

Antimycobacterial harms were common in patients living with NTM infections who received outpatient antimycobacterial treatment, where therapy was discontinued in over one-in-three patients. Pre-specified ADEs, or AKI, CDI, and thrombocytopenia, were less common than other ADEs that result in treatment discontinuation. Patients who received regimens containing an oxazolidinone or systemic aminoglycoside had a higher odds of developing AKI, CDI, or thrombocytopenia when compared with those who received other antimycobacterial classes. Additionally, patients who received an all-oral regimen were less likely to experience harm. ADEs will generally occur near the first month of outpatient treatment. Intensified monitoring and/or use of more tolerable antimycobacterial regimens early in treatment may be an appropriate approach to avoid harms. Future studies should evaluate early incorporation of more favorable agents into NTM treatment selection and subsequent impact on patient outcomes.
